# SATISFACTION WITH OUTDOOR ACTIVITIES AMONG LONG-TERM SERVICES AND SUPPORTS RECIPIENTS

**DOI:** 10.1093/geroni/igz038.1571

**Published:** 2019-11-08

**Authors:** Justine S Sefcik, Karen Hirschman, Darina V Petrovsky, Nancy Hodgson, Mary Naylor

**Affiliations:** 1 University of Pennsylvania School of Nursing, Philadelphia, Pennsylvania, United States; 2 University of Pennsylvania, Philadelphia, Pennsylvania, United States

## Abstract

Being outdoors in nature has been associated with improved mental and physical health. There are no known studies exploring older adults’ satisfaction with outdoor activities at the start of long-term services and supports (LTSS; in nursing homes, assisted living, or at home). We examined characteristics of older adults receiving LTSS and factors associated with outdoor activities satisfaction. A secondary analysis was conducted of baseline data involving structured interviews with older adults new to LTSS (Health-Related Quality of Life: Elders in Long-Term Care; R01AG025524-05). Primary outcome was a single item on the satisfaction with outdoor activities (not at all satisfied to extremely satisfied). We conducted multivariable linear regression models controlling for the influence of the characteristics important to health-related quality of life (LTSS setting, gender, age, number of comorbidities, and sensory impairment [vision/hearing].) Among 356 people, the majority (59%) were satisfied with their outdoor activities. Of 339 participants with complete data, more depressive symptoms (higher Geriatric Depression Score; p<.001) and higher cognitive functioning (higher MMSE score; p=.038) were associated with lower ratings of satisfaction with outdoor activities. Higher self-rated physical health (p=.038) and more independence with activities of daily living (p=.017) were associated with greater satisfaction with outdoor activities. Due to the cross-sectional nature of this study it is difficult to determine causality; however, outdoor activity is important to people receiving LTSS. Interdisciplinary teams can work with older adults receiving LTSS to assess interest level with outdoor activities and create a person-centered plan to increase outdoor activity and satisfaction levels.

